# Have All of the Phytohormonal Properties of Melatonin Been Verified?

**DOI:** 10.3390/ijms25063550

**Published:** 2024-03-21

**Authors:** Woong June Park

**Affiliations:** Department of Molecular Biology, Dankook University, Cheonan-si 31116, Republic of Korea; parkwj@dku.edu; Tel.: +82-41-550-3481

**Keywords:** phytomelatonin, hormone, pleiotropic effects, receptor

## Abstract

Melatonin is a ubiquitous regulator in plants and performs a variety of physiological roles, including resistance to abiotic stress, regulation of growth and development, and enhancement of plant immunity. Melatonin exhibits the characteristics of a phytohormone with its pleiotropic effects, biosynthesis, conjugation, catabolism, effective concentration, and the shape and location of its dose–response curves. In addition, CAND2/PMTR1, a phytomelatonin receptor candidate belonging to the G protein-coupled receptors (GPCRs), supports the concept of melatonin as a phytohormone. However, the biochemistry of plant melatonin receptors needs to be further characterized. In particular, some of the experimental findings to date cannot be explained by known GPCR signaling mechanisms, so further studies are needed to explore the possibility of novel signaling mechanisms.

## 1. Introduction

Since melatonin (*N*-acetyl-5-methoxytrypamine) was found in plants in 1995 [1,2,3], increasing efforts have been focused on phytomelatonin research. I found more than 860 papers in the most recent 5 years through a web search with the combined key words “melatonin” and “plant” (https://pubmed.ncbi.nlm.nih.gov (accessed on 24 January 2024)). Interestingly, about 200 papers among them are reviews. This high number of review papers may indicate the great interest of the community but also not enough research verifying the action mechanism.

Melatonin is originally known as a neurohormone found in the bovine pineal gland that regulates circadian rhythm, sleep, and other physiological events in the animal system, but it is now recognized as a multifunctional regulator in diverse life groups including invertebrates, plants, and procaryotes [4]. In plants, melatonin is involved in diverse physiological events (Table 1), including the mitigation of abiotic stress and the regulation of growth and development [5]. Furthermore, melatonin strengthens plant immunity and protects plants against pathogens, supplying new possibilities for agricultural application [6,7,8].

Melatonin has strong antioxidant activity of its own [9], so it can protect plants simply by being present at the right concentration. This intrinsic antioxidant activity is due to the chemical properties of the melatonin molecule, so that plant protection by high concentrations of melatonin is a response independent of cellular signaling. However, during evolution, plants appear to have acquired additional ways of using this substance that have been present since the earliest days of life on Earth [6]. By perceiving melatonin as a signal and linking it to intracellular signal transduction processes, plants were able to achieve a higher level of regulation through the amplification or diversification of responses to signal detection and integration of signals from different signaling pathways.

**Table 1:** Examples of the pleiotropic effects of melatonin in plants.

Plants	Responses	Effective Concentration	References
*Arabidopsis*	Alleviation of cold stress	10, 30 µM	[10]
	Upregulation of stress and defense genes and others	1 mM	[11]
	Mediation of innate immunity against bacterial pathogens	20 µM	[12]
	Expression of *CBF/DREB1*s genes involved in stress response	50 µM	[13]
	Induction of nitric oxide and enhancement of innate immunity	20 µM	[14]
	Cell wall strengthening and callose accumulation against bacteria	50 µM	[15]
	Suppression of root meristem, auxin biosynthesis, and transport	100 µM–1 mM	[16]
	Repression of the floral transition by stabilizing DELLA proteins	0.5, 1.0 mM	[17]
	Improvement of iron deficiency tolerance	5 µM	[18]
	Inhibition of brassinosteroid synthesis and decrease in hypocotyl growth	0.1–1.0 mM	[19]
	Promotion of lateral root development (synergism with auxin)	50–300 µM	[20]
	Regulation of stomatal closure	0.1–80 µM	[21]
	Inhibition of seedling growth and regulation of abscisic acid homeostasis	100, 300 µM	[22]
	Induction of pathogenesis-related proteins and other defense genes	10 µM	[23]
	Activation of mitogen-activated protein kinases (MPK3, MPK6)	1 µM	[24]
Maize	Improvement of germination by priming seeds with melatonin	50, 100 µM	[25]
	Delay of leaf senescence and improvement of antioxidant defense	25–75 µM	[26]
	Induction of resistance to a fungal pathogen, *Fusarium graminearum*	50–400 µM	[27]
	Enhancement of thermotolerance through modulation of antioxidant defense	10–70 µM	[28]
	Increase in drought stress tolerance	0.25–1.0 mM	[29]
Rice	Mitigation of cold-stress-induced reactive oxygen species (ROS) accumulation	20, 100 µM	[30]
	Regulation of root architecture and modulation of auxin response	10–50 µM	[31]
	Suppression of a pathogenic bacterial growth in rice	200 µg/mL	[32]
	Improvement of resistance to rice stripe virus	0.1–10 µM	[33]
	Reduction of fluoride uptake and toxicity	20 µM	[34]
	Broad-spectrum antifungal activity	0.1–10 mM	[35]
Soybean	Enhancement of growth and resistance to abiotic stress	50, 100 µM	[36]
	Activation of auxin biosynthesis and signal transduction	20 µM	[37]
	Alleviation of salt-alkali stress by reducing oxidative damage of DNA	300 µM	[38]
	Mitigation of arsenate stress	100 µM	[39]
Tomato	Promotion of adventitious root development	50 µM	[40]
	Improvement of tomato fruit quality and more ascorbic acid and lycopene	0.1 mM	[41]
	Mitigation of acid rain stress and modulation of leaf ultrastructure	50–250 µM	[42]
	Acclimation to a combination of abiotic stresses	100 µM	[43]
	Alleviation of photosynthetic apparatus under cold stress	5–250 µM	[44]
	Promotion of salicylic acid and nitric oxide accumulation and viral resistance	50–400 µM	[45]
	Improvement of cadmium tolerance	100 µM	[46]
	Delay of leaf senescence in darkness	250 µM	[47]
	Alleviation of heat-indued damage by balancing redox homeostasis	100 µM	[48]
	Improvement of cold tolerance	100 µM	[49]
	Alleviation of nickel toxicity	100 µM	[50]
	Ethylene-dependent enhancement of carotenoid biosynthesis	50 µM	[51]
	Increase in the resistance to the fungal pathogen *Botrytis cinerea*	1–100 µM	[52]
Wheat	Mitigation of salt stress through modulation of polyamine metabolism	1 µM	[53]
	Increase in photosynthetic capacity and salt tolerance	100 µM	[54]
	Enhancement of seed germination under salt stress	50–250 µM	[55]
	Reduction of chromium uptake and toxicity	1, 2 mM	[56]

Because melatonin has existed as a biomolecule since the dawn of evolution, its role in plants is complex, ranging from simple chemical action to receptor-mediated signaling. While this situation is advantageous for plants, it creates considerable confusion for melatonin researchers. The discovery of the melatonin receptor in plants [21] has led to great advances in understanding how melatonin works as a signal molecule and leads to the suggestion of melatonin as a new phytohormone [57,58], but the details of the action mechanism still remain poorly understood.

This review examines the melatonin responses in plants and aims to identify which of these are characteristic of phytohormone responses. It examines the effective concentration, biosynthesis, catabolism, transport, and dose–response curves of melatonin in plants and compares them with those of other phytohormones. Additionally, this paper reviews the current state of knowledge regarding the molecular biology and biochemistry of the plant melatonin receptor. This review examines the adequacy of available information in explaining the function of the melatonin receptor and discusses future works.

## 2. Pleiotropy

A characteristic of phytohormones is their pleiotropic action. The pleiotropy of phytohormone action is probably one of the reasons that it is possible to regulate a wide range of physiological responses with a limited number of phytohormones. In plants, the effects of melatonin are pleiotropic (Table 1).

The most widely accepted effect of melatonin in plants is its activity against oxidative stress. While melatonin itself is a potent antioxidant, it has the interesting property of inducing the expression of genes that alleviate oxidative stress. Melatonin is also known to interact with reactive oxygen species (ROS) and reactive nitrogen species (RNS) [6]. Not only against oxidative stress but also against diverse environmental stresses, e.g., salt, heat, drought, heavy metal, and strong light, melatonin protects plants [59]. Initially, melatonin attracted attention for its alleviating effects on abiotic stress, but now its protective roles against biotic stress are being illustrated. Melatonin reduces damage caused by viral, bacterial, and fungal infections in plants [7], giving the possibility to protect plants using melatonin as a defense stimulator [60,61,62]. Melatonin also affects plant growth and development, including root development, hypocotyl growth, germination, flowering, and parthenocarpy [57]. In many cases, these pleiotropic effects of melatonin do not occur directly but through a network of interactions with other phytohormones, including auxin, gibberellin (GA), cytokinin, abscisic acid (ABA), ethylene, brassinosteroids, salicylic acid, and jasmonic acid [6,63,64]. Melatonin and other phytohormones seem to share their signaling pathways very often. Melatonin can also change the concentration of other phytohormones, and the inverse has been known, too [64]. Interestingly, melatonin increases the concentration of itself [65]. All of melatonin’s effects concerning abiotic stress [5,6,66,67,68,69,70], biotic stress [6,7,8,71,72], and growth and development [5,66,69,70] have been extensively reviewed in recent years.

## 3. Biosynthesis, Conjugation, and Catabolism

Melatonin is biosynthesized and catabolized like other phytohormones, and its metabolic pathways consist of a regulatory network together with other phytohormones [64]. Melatonin is synthesized in plants from tryptophan (Trp) mainly via trypamine, serotonin, and *N*-acetylserotonin [66,73]. However, under some environmental stresses, melatonin is produced via alternative pathways using 5-hydroxytryptophan or 5-methoxytryptophan or both as intermediates. Indole-3-acetic acid (IAA), the representative auxin with an indole ring like melatonin, is essentially produced from Trp via indole pyruvic acid in *Arabidopsis* [74]. In parallel, several biosynthetic pathways to produce IAA have been suggested in diverse plants, including even a Trp-independent pathway based on the increase in IAA in the Trp-deficient maize mutant *orange pericarp* [75]. Melatonin and auxin share some intermediates, and this is one reason for the close relationship between melatonin and auxin [37].

The biosynthesis of melatonin is a part of the regulatory network. For example, ABA stimulates melatonin biosynthesis in watermelon by inducing gene expression of *ClASMT* encoding *N*-acetylserotonin methyltransferase, *ClCOMT* encoding caffeic acid *O*-methyltransferase, and *ClSNAT* encoding serotonin *N*-acetyltransferase [76].

To meet the physiological demands by optimizing hormone levels, plants have evolved sophisticated regulatory mechanisms of biosynthesis, catabolism, and conjugation. Melatonin participates in the regulation of homeostasis of other hormones in various modes. When plants experience water stress, ABA levels sharply rise to cope with the drought by closing stomata [77]. The increased ABA is reduced through degradation and conjugation [77]. In contrast to the cooperative effect of melatonin and ABA on plant responses to environmental stress, melatonin contributes to the maintenance of hormonal balance in *Malus* plants by decreasing the expression of the *NECD* gene encoding 9-cis-epoxycarotenoid dioxygenase, which is involved in ABA biosynthesis, or by promoting the expression of enzymes that catabolize ABA [68,78]. Melatonin also affects GA biosynthesis. Active GA homologues are produced by GA-3 oxidase, and the increased hormone is inactivated by GA-2 oxidase [79]. Melatonin regulates this fine-tuning of GA biosynthesis [80], suggesting a complex network in the regulation of hormone levels.

Melatonin promotes ethylene biosynthesis during tomato fruit ripening by inducing the expression of both 1-aminocyclopropane-1-carboxylic acid (ACC) synthase and ACC oxidase, which are involved in ethylene biosynthesis [51]. In this case, the apparent phenotypic change is fruit ripening, but the direct effect of melatonin is on ethylene biosynthesis; therefore, the observed effect of melatonin is indirect. These results suggest that mechanistic studies are needed to understand the action of melatonin. In the ethylene biosynthesis described above, there is a lack of information on how melatonin is perceived and linked to gene expression, although it is known that a MYB transcription factor binds directly to the promoter of the ACC synthase gene in another plant, grapevine [81]. Melatonin also affects the biosynthesis of melatonin itself. Exogenous melatonin increased endogenous melatonin levels in cassava [82] and maize [83], suggesting that self-stimulatory mechanisms may operate under certain circumstances.

Not only biosynthesis, but also conjugation and catabolism, are important tools to regulate hormone levels. All phytohormones are active in free form and lose their activity when conjugated to sugars or amino acids, except jasmonic acid, which is only active when conjugated to isoleucine [84]. Conjugation of melatonin was expected [85], but it has not yet been demonstrated in plants. When melatonin encounters hydroxyl radicals, melatonin can be chemically oxidized. In addition, melatonin is also enzymatically oxidized by melatonin 2-hydroylase [86] and melatonin 3-hydroxylase [87] to 2-hydroxy- melatonin and 3-hydroxymelatonin, respectively. Catabolism generally inactivates phytohormones. 2-oxoglutarate-dependent Fe(II) dioxygenase (DIOXYGENASE FOR AUXIN OXIDATION; DAO) oxidizes IAA to 2-oxindole-3-acetic acid (oxIAA) [88]. C26 hydroxylase (encoded by *BAS1*) and some other enzymes inactivate brassinosteroids [89]. GA2-oxidase inactivates GA [79]. Interestingly, unlike the oxidation products of other hormones, oxidized melatonin still exhibits antioxidant activity, although the activity is reduced to about 50%.

Multiple crosstalk among the regulatory mechanisms of phytohormone levels is an important tool to harmonize the diverse responses to cope with complicated situations for efficient survival. Melatonin, like other phytohormones, plays a role in this regulatory network, although the detailed mechanism remains to be elucidated.

## 4. Transport

Transport is an important means of regulating the local distribution of plant hormones. In particular, the polar transport of auxin by influx and efflux carriers is of great importance for the establishment of polarity during plant development. Fine-tuning the direction of auxin transport by redistributing efflux carriers (PINs) during development is an efficient way to establish a new axis [90]. Strigolactone moves from root to shoot and regulates apical dominance by suppressing axillary bud growth [91]. Melatonin transport is still poorly understood. Melatonin supplied to maize roots accumulates in the leaves, and the amount of accumulated melatonin decreases as the stomata closes [92], so it appears that melatonin moves along the transpiration stream in the xylem, but the details are unknown.

## 5. Dose Relationships

### 5.1. Effective Concentrations

Hormones are regulators that work at low concentrations, but the question is what range of concentrations is meant by low in this case. To see if the concentrations at which melatonin works are within the range of phytohormones, the working concentrations of other phytohormones are checked as well as that of melatonin. Auxin shows activity in the range of 0.1–10 µM to promote cell elongation in maize coleoptiles [93]. Cytokinin suppresses *Arabidopsis* root growth in the range of 0.01–10 µM [94]. Melatonin shows activity on stomatal closure in the range of 0.1–40 µM [21]. However, some effects of melatonin appear at much higher concentrations, sometimes even higher than 1 mM [95,96]. These differences in effective concentrations of melatonin for different effects could be due to whether the effects are via receptor signaling or not. Melatonin is a powerful antioxidant and can directly counteract oxidants generated during plant stress without the need for receptors at high concentrations.

### 5.2. Patterns of Dose–Response Curves

In phytohormone science, “dose–response” generally means “concentration gradient response”. Numerically, the “hormone dose” can be assumed to be “the concentration of a given hormone” times “the duration of the treatment”, as the light fluence is calculated as the multiplication of “photons per unit area” and “the duration time”. However, such an assumption is physiologically inappropriate for phytohormones, because the treatment with 1 µM IAA for 10 h and that with 10 µM IAA for 1 h gave qualitatively and quantitatively different results in stem growth [93]. Therefore, concentration gradient response curves obtained under the same experimental conditions are generally accepted as dose–response curves for phytohormones.

Because the binding of a phytohormone to its receptor is an equilibrium between bound and unbound states of the molecules, most of the dose–response curves show changes over several orders of magnitude of the hormone concentrations [97]. Because dose–response curves of phytohormones reflect the status of receptor and changes in signal transduction, observation of dose–response curves is very useful to obtain information about the hormone action.

As shown by the dose–response curves for phytohormones, responses vary depending on the level of hormone, providing an efficient way to regulate hormone action (Table 1). They typically show a saturating sigmoid or inverse sigmoid curve when the hormone response is plotted against hormone concentration [98,99,100] or a bell-shaped characteristic [93,101] where the response peaks at a certain concentration and then declines. If the hormone response is suppressive, the bell-shaped dose–response curve can also be expressed as a U-shaped curve [21] (Table 2). The shape of dose–response curves for the same hormone can vary depending on the type of response, tissue, organ, plant species, and many other conditions.

The dose–response curve of melatonin for stomatal closure is U-shaped [21], but the curve for *Arabidopsis* root growth is inverse sigmoidal [16]. Because the dose–response curve of melatonin over 10^−8^ M to 10^−4^ M for coleoptile and root growth in some monocot plants was not simply bell-shaped and was even somewhat inhibitory at high concentrations [102], the character was interpreted as hormetic [103].

**Table 2:** Types of dose–response curves for some phytohormones.

Dose–Response	Hormone	Response	References
Sigmoidal			
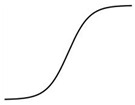	Auxin	Elongation of maize coleoptiles and pea stems	[98]
		Petiole elongation in *Ranunculus sceleratus*	[104]
	Gibberellin	Leaf elongation in the dwarf mutants of barley	[99]
	Cytokinin	Amaranthin accumulation	[105]
	Strigolactone	Germination in some parasite plants	[106]
		
Inverse sigmoidal			
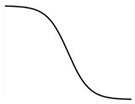	Auxin	Root growth in *Arabidopsis*	[107]
	Cytokinin	Root growth in *Arabidopsis*	[94]
	Abscisic acid	Germination in *Arabidopsis*	[108]
	Brassinosteroid	Growth of etiolated pea seedling	[109]
	Melatonin	Root growth in *Arabidopsis*	[16]
		
Bell-shaped			
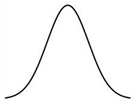	Auxin	Maize coleoptile elongation by IAA and 4-Cl-IAA	[101]
		Pea epicotyl protoplast swelling	[110]
		Maize coleoptile elongation	[93,111]
	Strigolactone	Seed germination in some *Striga* plants	[112]
	Salicylic acid	PR1 accumulation in tobacco cell culture	[113]
	Melatonin	The maximum quantum yield of photosystem II (Fv/Fm)	[114]
U-shaped			
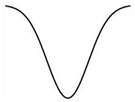	Melatonin	Stomatal closure in *Arabidopsis* Malondialdehyde (MDA) content	[21,114]

### 5.3. Changes in Dose–Response Curves and Regulatory Implications

**Figure 1:**
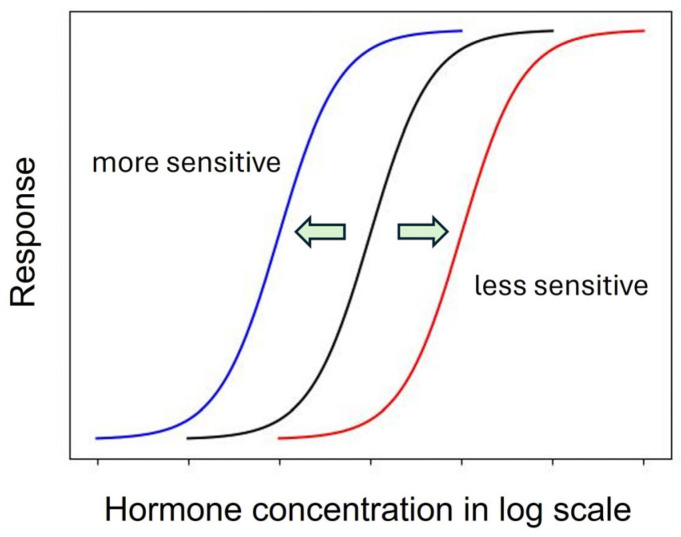
Dose–response curves of a phytohormone reveal the sensitivity change reflecting the ligand affinity of the receptor. Arrows indicate the direction of shift of the original dose-response curve (black).

As described above, the shape of the dose–response curve is important, but another equally important characteristic is the location of the dose–response curve [97]. A dose–response curve on the left indicates a more sensitive response than a dose–response curve on the right (Figure 1).

A leftward shift of a dose–response curve has been suggested to indicate an increased affinity of the receptor for its ligand [115]. However, the rightward shift of the dose–response curve of root growth to auxin in the *aux1* mutant [107], which has a defective auxin influx carrier, suggests that some cause other than a change in receptor affinity may induce the leftward or rightward shift of the dose–response curve.

Generally, this type of change is described by the term “sensitivity change”, which has a broad meaning and includes changes in affinity, receptivity (changes in the number of receptors), endogenous hormone levels, or any other physiological changes that affect hormone responses [115]. The dose–response curves of maize coleoptiles to auxin were different when treated with auxin 0.5 h or 2.5 h after tissue excision, indicating a time-dependent change in auxin sensitivity [116]. Furthermore, in a semiaquatic plant, *Ranunculus sceleratus*, petiole elongated auxin dependently, and the dose–response curve of the growth was shifted to the left by ethylene [104]. Interestingly, ethylene desensitization is mediated by a gene family, *ARGOS*, whose expression is induced by auxin in *Arabidopsis* [117]. Trewavas (1983) recognized such a great potential to regulate hormonal action by modulating sensitivity without changing phytohormone levels [75]. However, the regulation mechanism of phytohormone sensitivity remains largely unexplored. Despite its importance, the sensitivity regulation of melatonin action also remains to be investigated.

## 6. Receptors

### 6.1. Finding of Receptors and Disputes

Probably one of the most important proofs that a bioactive substance is a hormone is the presence of its receptor. Even after it was widely recognized that melatonin exists in plants and has a wide range of bioactivities, how plants perceive it remained a mystery. Therefore, the first paper suggesting that PMTR1 in *Arabidopsis* is the melatonin receptor [21] has received a lot of attention. PMTR1 was a CAND2 (candidate GPCR2) identified in *Arabidopsis* from previous studies, but its function was unknown [118]. The CAND2 protein (Q94AH1.1) consists of 300 amino acid residues, has a molecular weight of 34.1 kDa and an isoelectric point (pI) of 6.38, and contains seven transmembrane domains. Sequence analysis indicated that this protein belongs to a branch of GPCRs. Because the animal melatonin receptors, M1 and M2, are high-affinity GPCRs, the idea that the plant melatonin receptor is also a GPCR was intriguing. Since the first report, proteins with similarity to CAND2/PMTR1 have been identified in *Panax notoginseng* [119], *Nicotiana benthamiana* [120], *Medicago sativa* [121], *Zea mays* [122], and *Gossypium hirsutum* [123]. In CAND2/PMTR1 knock-out mutants or overexpressed plants, stomatal closure [21,124], immunity [119,120], osmotic stress tolerance [125], salt and osmotic stress tolerance [121,122] and mitochondrial gene expression [126] were affected, supporting the idea that the CAND2/PMTR1 is the actual phytomelatonin receptor. However, some researchers questioned the conclusion that CAND2/PMTR1 is a receptor for phytomelatonin because they obtained different results in *Arabidopsis* [127], whereas CAND2/PMTR1 was first reported to be the receptor for phytomelatonin [21].

The workers double-checking the first report observed slight differences in the localization of CAND2/PMTR1-mCherry and FLS-2-GFP (as a plasma membrane marker), melatonin-induced MAPK (MPK3/6) activation and several gene expressions in *cand2* mutants, and MPK3/6 activation in the *gpa1* mutant [127]. The first report showed the localization of CAND2/PMTR1-YFP and YFP (as a cytoplasmic marker) in separate cells without the use of a plasma membrane maker [21]. Unfortunately, the location of YFP was confusingly similar to that of CAND2/PMTR1-YFP [21], although the authors interpreted the result as confirming the plasma membrane localization of CAND2/PMTR1. Later, it was shown that the localization of GFP-PMTR1 and PIP2-mCherry (a plasma membrane marker) coincided with another group [119]. The plasma membrane localization of CAND2/PMTR1 was also confirmed in alfalfa [121].

The next argument was the activation of MPK3/6 by melatonin in the *cand2-1* and *cand 2-2* mutants [127]. The *cand2-1* was used in the first report to show the aberrations in the stomatal closure and ion fluxes regulated by melatonin [21]. To investigate this point, the researchers who double-checked the first report measured the transcript level of CAND2 in *cand2-1* and *cand2-2* and found that the CAND2 mRNA accumulation was abolished in the *cand2-2* but not in the *cand2-1* [127], which was used to obtain evidence for the receptor function of CAND2/PMTR1 [21]. However, they did not check the CAND2/PMTR1 protein level. Because the transcript level does not necessarily correspond to the protein level, this question remains open.

Another argument was that melatonin-induced gene expression and MPK3/6 activation were normal in the *gpa1* mutant [127]. GPA1 encodes Gα, which is part of a heterotrimeric GTP-binding protein that interacts with GPCRs and is known to play a key role in signal transduction. Because CAND2/PMTR1 was identified as a GPCR, the normal function of melatonin in the *gpa1* mutant seemed to make no sense, casting doubt on CAND2/PMTR1 as the melatonin receptor. However, melatonin-induced activation of MPK3/6 was impaired in *pmtr1* mutant but was normal in the *gpa1-4* mutant [119], strongly suggesting the involvement of another signaling pathway that is GPA1-independent. In contrast, melatonin-induced stomatal closure was aberrant in the *gpa1-4* mutant [119].

The evidence collected to date suggests that CAND2/PMTR1 acts as a receptor for phytomelatonin. However, if one were to add another question, it would be about the biological benefits of localizing the melatonin receptor to the plasma membrane. What would be the advantages of signal perception at the cell surface for melatonin, which easily passes through membranes?

### 6.2. Biochemistry of the Receptors

The main feature of a hormone receptor is binding to its ligand. The binding can be biochemically described following a saturating competitive binding assay. Several papers on CAND2/PMTR1 included the binding assay results and reported that the K_d_ of CAND2/TMPR1 was about 0.73 nM in *Arabidopsis* [37] and about 1.026 nM (with B_max_ 0.93 pmol/6 nmol protein) in alfalfa [128]. Another paper reported the EC_50_ value of 47.8 nM in maize [122]. Unfortunately, the procedures for the binding assay and the results were described only briefly. Thus, it remains unclear how the melatonin was labeled, whether the equilibrium was properly achieved, whether there was adequate competition with unlabeled melatonin, how the specific binding was estimated, and how K_d_ and B_max_ were determined. Because the molecular weight of the CAND2/PMTR1 protein is known, it should be possible to calculate the number of ligand binding sites on the receptor from B_max_, but the experimental results were presented too simplistically to make the necessary calculations.

One paper presented a nice saturation curve of melatonin binding to CAND2/PMTR1 as evidence for ligand binding to the candidate receptor in cassava [128]. The shape of the saturation curve looked similar to that observed for other hormone binding but was in fact unique when the fold change of melatonin from basal to saturation level was considered. In their hands, the binding was saturated when melatonin was increased from 0.5 to 1.5 nmol in the presence of 20 mmol of purified MePMTR1 [128]. In general, saturation of ligand binding to a hormone receptor occurs over at least two or more orders of magnitude of increase in ligand concentration. Therefore, it is very difficult to observe the saturation of ligand binding with only the labeled ligand due to the difficulty of preparing high concentrations of labeled ligand. Instead, the binding characteristics of a ligand to its receptor are measured by preparing a certain concentration of labeled ligand and allowing it to compete with various concentrations of unlabeled ligand for binding to the receptor. The competitor ligand decreases the measured indicator of the labeled ligand, such as radioactivity, in a concentration-dependent manner. However, the specific activity of the ligand mixture provides the actual amount of increasing ligand binding to the receptor, including both the labeled and unlabeled ligand. The binding saturation occurs over several orders of magnitude of the ligand. Unfortunately, the description of the unique aspect of melatonin binding to MePMTR1 in a narrow range was omitted in the paper [128].

The long debate over auxin-binding protein 1 (ABP1) [129,130,131,132,133,134] shows that hormone-binding proteins must be thoroughly validated before they can be accepted as receptors. After the first discovery of auxin binding to membranes [135], efforts were made to find proteins that specifically bind to auxin. A protein isolated from maize shoots, which have been extensively used to monitor auxin activities, showed specific auxin binding with a Kd value of 6 × 10^−8^ M [136]. Finally, the gene for the auxin-binding protein was cloned, and the molecular characteristics of ABP1 were revealed [137]. ABP1 had a molecular mass of 22 kDa and contained the endoplasmic reticulum retention signal KDEL, which could explain the fact that auxin binds to the endoplasmic reticulum, as reported in previous papers [138]. However, based on discrepancies between the dose–response curve of 1-naphthalene acetic acid (1-NAA; an artificial auxin) and the K_d_ value of ABP1, along with several other uncertainties, skeptical questions have been raised about the receptor function of ABP1, even calling it a red herring [129] or an outsider [131]. Further molecular biological and biochemical evidence supporting the physiological function of ABP1 has been presented and repeatedly rejected [134], leaving the role of ABP1 as an auxin receptor uncertain. As seen in the ABP1 debates, further efforts are needed to confirm the physiological function of CAND2/PMTR1 as a phytomelatonin receptor.

To establish the physiologically relevant melatonin receptor function of CAND2/PMTR1, the first step would be an elaborated biochemical description of the binding properties, followed by a good match between the biochemical properties of the hormone-binding protein and the molecular physiological responses induced by melatonin. With regard to this point, I still have questions about the reason for the difference between the melatonin dose–response and the melatonin binding characteristics of CAND2/PMTR1; for example, stomatal closure showed maximum activity at 1 to 10 µM melatonin, but melatonin binding was already saturated at 100 nM [21]. The next step would be to determine whether the binding of melatonin alters the conformation of CAND2/PMTR1 and whether these changes are associated with subsequent signaling. In particular, the interaction between CAND2/PMTR1 and GPA1 is important because there are some conflicting results. Scrutinizing the action of GPA1 may provide new clues that lead us to a different signaling pathway or even an unexpected new receptor.

## 7. Perspectives

**Figure 2:**
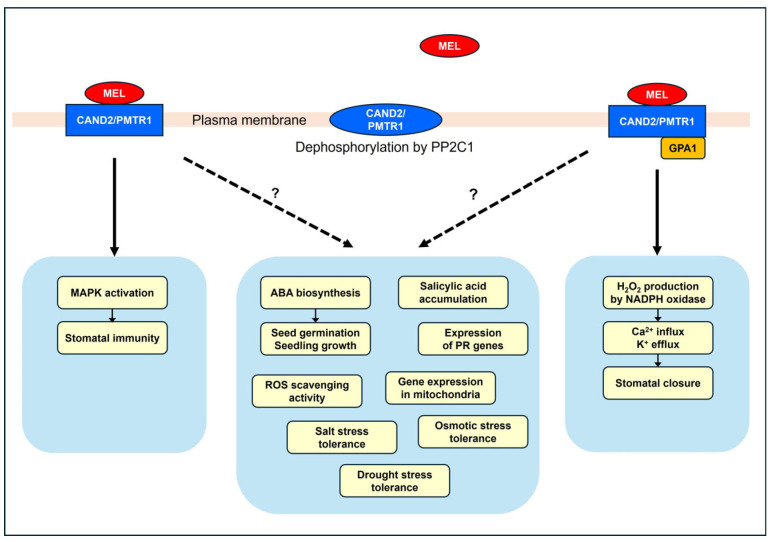
Melatonin responses regulated by CAND2/PMTR1. MAPK activation occurs independently of GPA1 [119], while stomatal closure is dependent on GPA1 [119]. The mechanisms that regulate ABA biosynthesis [22], salicylic acid accumulation [120], expression of PR genes [120], mitochondrial gene expression [126], ROS scavenging activity [125], and tolerance to salt [123], drought [122], and osmotic [125] stress under the regulation of CAND2/PMTR1 are not yet fully understood. Dephosphorylation of CAND2/PMTR1 abolishes the binding of melatonin to CAND2/PMTR1 [128].

Phytomelatonin is biosynthesized and degraded in plants and induces a variety of responses. Some of the responses induced by low concentrations of melatonin show characteristics of hormone responses, while other responses to high concentrations of melatonin may be due to the chemical nature of melatonin. One of the most critical criteria for recognizing melatonin as a phytohormone is the existence of the receptor. CAND2/PMTR1 is currently considered the primary candidate for the phytomelatonin receptor. Mutations in CAND2/PMTR1 have been found to either abolish or reduce several melatonin-induced responses in plants, indicating its critical involvement in melatonin actions. However, there are melatonin responses that cannot be explained by the general action mode of the G protein-coupled receptors to which CAND2/PMTR1 belongs. There may be signaling pathways of phytomelatonin that are dependent on GPA1, and others that are independent of it (Figure 2). Furthermore, the signaling mechanisms for many other physiological responses regulated by CAND2/PMTR1 remain unclear. To establish CAND2/PMTR1 as a receptor for phytomelatonin, it is necessary to thoroughly characterize its biochemical properties. Then, the mechanisms of signal transduction that induce the molecular physiological responses to melatonin must be elucidated. The melatonin responses in plants that are not accounted for by CAND2/PMTR1 may be attributed to the chemical properties of melatonin itself. However, it is also possible that plants have a distinct signaling process that differs from the classical GPCR signaling. It is also possible that plants even have another type of phytomelatonin receptor in addition to CAND2/PMTR1.
